# Trends of Components of the Metabolic Syndrome in German First Graders Throughout 10 Years: The PEP Family Heart Study

**DOI:** 10.1155/2012/231962

**Published:** 2012-07-05

**Authors:** Gerda-Maria Haas, Thomas Bertsch, Peter Schwandt

**Affiliations:** ^1^Arteriosklerose-Präventions-Institut, 81477 München, Germany; ^2^Institute of Clinical Chemistry, Klinikum Nürnberg, 90419 Nürnberg, Germany; ^3^Ludwig-Maximilians-Universität München, 80539 München, Germany

## Abstract

Although childhood overweight and obesity are increasing worldwide, some countries report trends for stabilization. However, the trend for the potentially atherogenic components of the metabolic syndrome (MetS) in children and adolescents is not well understood. Therefore, the purpose of this study was to analyze the trend of the five components of over 10 years in 2228 first graders aged 6 years. Waist circumference (WC) remained mainly unchanged between 1994 and 2003 whereas the other four components continuously decreased. In boys and girls mean values of triglycerides (−25.9% and −28.6%, resp.), HDL cholesterol (−19.8% and −23.4%, resp.), fasting glucose (−7.3% and −9%, resp.), systolic (−3.8% and −4.1%, resp.), and diastolic (−10.2% and −9.7%, resp.) blood pressure significantly decreased. Whereas the prevalence of abdominal adiposity was stable at baseline and after 10 years (−1% in boys and +2% in girls), the prevalence of hypertension, hypertriglyceridemia, low HDL-C, and glucose was very low without any trend.

## 1. Introduction 

 Overweight, obesity, and the metabolic syndrome (MetS) are an emerging health problem in industrialized and developing countries [1, 2]. Ethnic disparities are reported regarding the prevalence of MetS, which was four times higher in Iranian than in German adolescents as well as regarding single components in terms of considerably higher prevalence of dyslipidemia in Brazilian and Iranian compared with German youths [3, 4]. 

 Secular trends of childhood overweight and obesity in terms of increased body mass index (BMI) are heterogeneous in different countries with a large variation across countries [5–7]. However, BMI is not a component of the metabolic syndrome (MetS) according to the International Diabetes Federation (IDF) defining central obesity for children and adolescents as waist circumference (WC) at or above the 90th percentile [8]. Increasing prevalence of central adiposity indicates increasing cardiovascular risk [9, 10]. Furthermore, early elementary school years are a critical period for increases in obesity prevalence sharply increasing from 10.4% to 19.6% below and above age six years [11]. Therefore, the purpose of this study was to assess the trend of the five single components over 10 years in a large sample of six-year-old first graders.

## 2. Material and Methods

 We investigated 2228 German first graders (1116 boys and 1112 girls, median age 6.0 years) who participated in yearly cross-sectional surveys between 1994 and 2003. Continuously trained research assistants measured waist circumference (WC), systolic and diastolic blood pressure (BP), fasting triglycerides (TG), high-density cholesterol (HDL-C), and fasting plasma glucose (FPG) as previously described [12]. We used the IDF cut-offs for the five MetS components in terms of WC ≥ 90th percentile, SBP ≥ 130 or DBP ≥ 85 mm Hg, TG ≥ 1.7 mmol/L, HDL-C ≤ 1.03 mmol/L and glucose ≥ 5.6 mmol/L [8].

 For statistical analyses, we used SPSS 18.0 and the Kruskal-Wallis rank test for multisample ordinal comparisons, the Wilcoxon rank-sum test for pairwise comparisons and for linear trends across time ANOVA. *P* < 0.05 was considered significant.

## 3. Results and Discussion

 As presented in Table 1 mean values of waist circumference (−1% in boys and +2% in girls) and of body mass index (+0.6 in boys +1.9% in girls) remained stable between 1994 and 2003. This is consistent with the stabilization of BMI between 1999 and 2010 among children and adolescents in USA, UK, and Sweden [13–15]. In Stockholm, the prevalence of obesity differed in 10-year-old boys by +0.6% and in girls by −1.6% between 1999 and 2003 which is comparable with the urban area of Nuremberg [15]. “Mean WC greatly increased among US children between 1988–1994 and 1999–2004” [13]. However, among 2–5-year-old children the increase was +2.3% in boys and +1.6% in girls and among 6–11-year-old children WC increased by +4.2% in boys and +4.9% in girls. We found a slight decrease by −1% in boys respectively, a slight increase by +2% in girls from 1994 to 2003 among 6-year-old first graders. These small differences between the monocentric PEP Family Heart Study using the same staff, location, and equipment and the NHANES design, which includes different ethnicities, are remarkable though different measuring points for WC were used (midway between lowest rib and iliac crest versus high point of the iliac crest). Furthermore, periods of assessment are different as well as age (6-year olds and ages 6–11 years). Among Australian children the mean WC z-score in 2–16-year-old children increased more in girls than in boys at a faster rate than BMI between 1985 and 2007 [9]. In Spanish adolescents and in British adolescents and children aged 2–5 years WC increased significantly in both genders from 1995 to 2000–2002, respectively, from 1977 to 1997 [16–18]. A recent review compares mean values of WC in 6-year-old children from 11 countries [19]. 

 However, the mean values of the other four components of MetS significantly decreased (Table 1). The strongest decrease was observed for mean TG (−25.9% in boys, −28.6% in girls) and HDL-C (−19.8% in boys, −23.4% in girls). DBP (−10.2% in boys, −9.7% in girls) and SBP (−3.8% in boys, −4.1% in girls) decreased less, and fasting glucose (−7.3% in boys, −9% in girls) was available only for the last 4 years. Because all measurements were performed at entry in the PEP Family Heart Study before any lifestyle advice was implemented, we suggest that the decrease of the nonanthropometric risk variables might be due to secular changes. 

 We registered no clear trend of the prevalence of four MetS components between 1994 and 2003 among 6-year-old children except for glucose for the last four years of the study, however unavailable values from the years before (Table 2). The prevalence of abdominal obesity decreased in boys (−0.5% from 9.6% at baseline) but increased in girls (+4.7% from 6.7% at baseline). In children with abdominal obesity the interrelationship with hypertension (*χ* ^2^ = 29.3, *P* < 0.001) and hypertriglyceridemia (*χ* ^2^ = 4.6, *P* = 0.013) was significant, and the interrelationship between hypertriglyceridemia and low HDL-C was (*χ* ^2^ = 39.8, *P* < 0.001). In 1994 the prevalence of raised triglycerides (4.2% in boys and 4.8% in girls) and of low HDL-C (1.8% in boys and 0.7% in girls) was far less than 21.5%, respectively, 18.7% in 5–17.9-year-old obese children in European Union in 2006 [14]. The highest prevalence of hypertension in 6-year-old was 3.6% in boys and 6.3% in girls in 1997 and was considerably lower than in European Union in 2006. A very large European multicenter study in 26 008 overweight children reported that 50% had at least one cardiovascular risk factor related to the degree of overweight in terms of high BMI [20]. Since only 5.9% of the 12.6-year-old children were normal weight, the risk factor profiles cannot be compared with mainly normal-weight first graders of the current study, which however focused on WC as the major MetS component. Furthermore, studies from Germany reporting secular trends of BMI did not present nonanthropometric risk variables [21–24].

 This is the first study documenting stable mean values of waist circumference and decreasing trends for the mean other four components of the metabolic syndrome over 10 years in first graders at the critical age of six years. However, it is unclear whether stable WC reflects a plateau between 1994 and 2003 and whether continuous decrease of MetS components mean values indicates leveling off in cardiovascular risk. Further strength of this monocentric study is constancy of staff, location, procedures, methods, and equipment and that all first graders lived in the same PEP families over ten years. A limitation of this study is the low prevalence of MetS components in this large cohort, which only allows a trend analysis of mean values regarding the four nonanthropometric variables. A further limitation is the relatively small sample size of 2228 first graders to detect a 10% difference between changes of prevalence [13]. Furthermore, the percentage of children with central obesity (≥90th percentile) was too small for categorizing the participants in prevalence groups of high WC (≥85th, ≥95th, ≥97th, and ≥99th percentiles) as has been done for Californian adolescents. This very large study (8.3 million multiethnic adolescents) was powered to demonstrate declining prevalence of high BMI for some groups but not for Indian and black girls [25]. 

## 4. Conclusions 

 This is the first study presenting ten years' trends of the mean values of the five components of the metabolic syndrome according to the definition of the International Diabetes Federation in first graders. Mean values of waist circumference as well as the prevalence of abdominal adiposity remained stable in 6-year-old children between 1994 and 2003. But the prevalence of hypertension, hypertriglyceridemia, low HDL-C, and hyperglycemia were low and inconsistent though mean values of blood pressure, triglycerides, HDL-cholesterol, and glucose decreased continuously in both genders.

Longitudinal studies are needed to confirm these disparate trends. 

## Figures and Tables

**Table 1:** Characteristics and components of the metabolic syndrome in 2228 first graders between 1994 and 2003.

	1994	1995	1996	1997	1998	1999	2000	2001	2002	2003
	1,116 boys; mean (SD)									

Mean age (SD)	6.4 (0.5)	6.4 (0.5)	6.4 (0.5)	6.6 (0.5)	6.5 (0.5)	6.4 (0.5)	6.4 (0.5)	6.4 (0.5)	6.4 (0.5)	6.3 (0.5)
Median	6.0	6.0	6.0	7.0	6.0	6.0	6.0	6.0	6.0	6.0
BMI (kg/m²)	15.8 (2.4)	15.6 (1.7)	16.0 (1.8)	16.2 (2.4)	16.4 (2.3)	15.8 (1.7)	15.6 (1.7)	16.1 (1.8)	16.0 (2.1)	15.9 (2.4)
WC (cm)	56.9 (5.6)	56.0 (4.8)	58.0^∗^ (4.7)	58.6 (6.8)	58.7^∗^ (5.7)	57.4 (4.2)	56.8 (4.0)	56.3 (4.7)	56.6 (4.8)	56.3 (6.1)
SBP mmHg (SD)	105.1 (10.0)	104.2 (8.7)	104.7 (8.9)	106.2 (9.2)	104.5 (10.3)	104.0 (6.8)	103.9 (8.6)	101.8^∗^ (7.8)	102.6^∗^ (6.7)	101.1^∗^ (7.7)
DBP mmHg (SD)	70.7 (8.4)	67.1^∗^ (7.1)	70.9 (9.0)	71.1 (6.8)	71.8 (7.8)	66.2^∗^ (7.1)	67.4^∗^ (7.5)	62.1^∗^ (7.1)	63.8^∗^ (7.0)	63.5^∗^ (5.7)
Triglycerides (mmol/L) (SD)	0.85 (0.42)	0.65^∗^ (0.28)	0.64^∗^ (0.22)	0.67 (0.21)	0.63^∗^ (0.16)	0.76^∗^ (0.33)	0.67^∗^ (0.25)	0.61^∗^ (0.26)	0.62^∗^ (0.23)	0.63^∗^ (0.23)
HDL-cholesterol (mmol/L) (SD)	1.73 (0.35)	1.55^∗^ (0.40)	1.51^∗^ (0.28)	1.60 (0.34)	1.57^∗^ (0.26)	1.39^∗^ (0.31)	1.37^∗^ (0.24)	1.47^∗^ (0.28)	1.47^∗^ (0.36)	1.54^∗^ (0.26)

	1,112 girls; mean (SD)									

Mean age (SD)	6.4 (0.5)	6.4 (0.5)	6.3 (0.5)	6.6 (0.5)	6.4 (0.5)	6.4 (0.5)	6.4 (0.5)	6.3 (0.4)	6.3 (0.5)	6.3 (0.5)
Median	6.0	6.0	6.0	7.0	6.0	6.0	6.0	6.0	6.0	6.0
BMI (kg/m²)	15.6 (2.4)	15.7 (2.2)	15.7 (1.7)	16.2^∗^ (2.4)	15.8 (1.3)	15.9 (2.2)	16.2^∗^ (2.0)	15.8 (1.8)	16.3^∗^ (2.6)	15.9 (1.9)
WC (cm)	55.0 (5.4)	55.4 (5.5)	56.4^∗^ (4.4)	58.8^∗^ (5.9)	56.4 (3.9)	57.3^∗^ (6.1)	57.1^∗^ (5.8)	55.5 (4.7)	56.9^∗^ (5.5)	56.1 (4.8)
SBP mmHg (SD)	105.2 (10.0)	104.1 (9.0)	103.5 (9.2)	104.3 (9.4)	105.0 (9.6)	104.0 (10.2)	104.2 (8.2)	101.5^∗^ (7.6)	102.9 (6.9)	100.9^∗^ (7.6)
DBP mmHg (SD)	71.0 (8.5)	67.1^∗^ (6.9)	70.0 (8.4)	71.8 (9.7)	71.9 (7.6)	66.0^∗^ (8.6)	67.5^∗^ (7.5)	63.7^∗^ (7.0)	64.5^∗^ (6.7)	64.1^∗^ (7.0)
Triglycerides (mmol/L) (SD)	0.91 (0.41)	0.71^∗^ (0.26)	0.73^∗^ (0.30)	0.68^∗^ (0.07)	0.88 (0.42)	0.74^∗^ (0.21)	0.82 (0.22)	0.73^∗^ (0.30)	0.73^∗^ (0.29)	0.65^∗^ (0.21)
HDL-cholesterol (mg/dL)	1.71 (0.35)	1.38^∗^ (0.36)	1.49^∗^ (0.34)	1.50 (0.47)	1.38^∗^ (0.47)	1.41^∗^ (0.27)	1.41^∗^ (0.30)	1.28^∗^ (0.36)	1.40^∗^ (0.29)	1.31^∗^ (0.21)

*P* < 0.05 compared with baseline values.

**Table 2:** Prevalence (%) of the five components of MetS according to the IDF definition in 2228 German first graders between 1994 and 2003.

Year of enrollment of first graders	WC ≥ 90th percentile	TG ≥ 1.7 mmol/L	HDL-C ≤ 1.03 mmol/L	SBP ≥ 130 mmHg DBP ≥ 85 mmHg
boys				

1994	9.6	4.2	1.8	0
1995	5.3	2.9	7.8	0
1996	12.1	0	5.6	0
1997	10.7	0	0	3.6
1998	19.1	0	0	2.1
1999	7.6	3.3	10.0	0
2000	6.7	0	8.3	0
2001	6.5	0	0	0
2002	6.8	0	10.8	0
2003	9.1	0	5.9	0

girls				

1994	6.7	4.8	0.7	0
1995	5.5	0	14.0	0
1996	8.3	0	5.2	1
1997	18.8	0	20.0	6.3
1998	6.5	8.3	8.3	0
1999	12.7	0	4.8	0
2000	10.8	0	9.4	0
2001	9.5	0	26.3	1.6
2002	11.3	0	7.7	0
2003	11.4	0	18.9	0

WC indicates waist circumference, TG: triglycerides, HDL-C: high-density lipoprotein cholesterol, SBP: systolic blood pressure, DBP: diastolic blood pressure.
